# Enhancing Collaboration and Integrated Vision on Health: Key Strategies for Addressing Knee Osteoarthritis

**DOI:** 10.5334/ijic.8969

**Published:** 2025-09-16

**Authors:** Melissa J. J. Voorn, Tim A. E. J. Boymans, Albère J. A. Köke, Marion A. C. de Mooij, Clemens G. M. Rommers, Jascha de Nooijer, Marielle J. E. B. Goossens, Ivan P. J. Huijnen, Jeanine A. M. C. F. Verbunt

**Affiliations:** 1 Faculty of Health, Medicine and Life Sciences, Department of Rehabilitation Medicine, Functioning, Participation & Rehabilitation, School for Public Health and Primary Care (CAPHRI), Maastricht University, Maastricht, The Netherlands; 2 Centre of Expertise in Rehabilitation and Audiology, Adelante, Hoensbroek, The Netherlands; 3 Maastricht university medical centre+, Department of Orthopaedics, Maastricht, the Netherlands; 4 Zuyd University of Applied Sciences, Heerlen, The Netherlands; 5 Department of Health Promotion, School of Health Professions Education, Faculty of Health, Medicine and Life Sciences, Maastricht University, Maastricht, The Netherlands

**Keywords:** interdisciplinary care, patient experience, knee osteoarthritis, integrated health perspective, stakeholder analysis, collaborative care, interdisciplinary training

## Abstract

**Background:**

Persons with knee osteoarthritis suffer from a prevalent chronic condition requiring comprehensive treatment approaches. To effectively cater to their needs, healthcare professionals must adopt an integrated health perspective and collaborate across disciplines. This study aimed to understand the viewpoints of patients and healthcare professionals and identify factors influencing collaborative care for persons with knee osteoarthritis.

**Methods:**

Semi-structured interviews were conducted with knee osteoarthritis persons and healthcare professionals involved in their care. Data analysis involved identifying common themes, patterns, and discrepancies among stakeholders. Interviews were coded using NVivo, and inductive analysis was employed to determine themes.

**Results:**

Nine healthcare professionals from various disciplines and eight patients with knee osteoarthritis participated. Five main themes emerged: diagnostics, communication, training conditions, integrated health perspective, and Perceived collaboration and support from healthcare professionals. Persons with knee osteoarthritis sought comprehensive diagnoses and expressed satisfaction. Healthcare professionals faced barriers like limited integrated health perspective and time constraints, stressing the need for interdisciplinary training and collaboration.

**Conclusion:**

Implementing an integrated healthcare vision is crucial for personalized care in chronic conditions like knee osteoarthritis. Continuous training and curriculum integration for healthcare professionals are necessary to prioritize integrated health perspectives and collaboration, thus better meeting the diverse needs of persons.

## Introduction

Knee osteoarthritis is a prevalent degenerative disorder characterized by joint pain and functional impairment [1]. The World Health Organization (WHO) has designated the period from 2021 to 2030 as the decade of healthy aging, emphasizing the importance of addressing conditions like osteoarthritis, which substantially impact ones functional abilities and overall quality of life [1]. Compared with the general population, persons with osteoarthritis exhibit nearly three times higher reporting of poor health and have double the likelihood of experiencing high psychological distress and severe pain [2]. As the most common cause of disability among the elderly, osteoarthritis profoundly limits their ability to perform daily tasks and activities [2].

Despite the chronic and incurable nature of osteoarthritis, general practitioners frequently refer patients to orthopedic surgeons, even though a Dutch study found that only 10% of patients in orthopedic practices had received all recommended non-surgical treatments before undergoing surgery [3,4]. This trend reflects a predominantly biomedical approach, focusing on addressing specific complaints such as pain, which may not effectively manage the complex challenges posed by chronic conditions like osteoarthritis [5]. This way of managing pain, which typically align with persons demands, fits for an acute health situation. But in case of a chronic incurable condition like osteoarthritis it may result in a battery of diagnostics and treatments, that most of the time will end, unfortunately, without the desired solution of pain relief. In this situation, an integral vision on health with a focus on optimizing functioning despite the existence of a chronic condition seems more appropriate. Consequently, patients often seek various healthcare solutions, including non-operative interventions and joint replacement surgery, with varying degrees of success [6,7]. Traditional measures of health status may inadequately capture the comprehensive impact of osteoarthritis on patients’ lives, particularly regarding functioning and participation in daily activities [8,9,10,11]. This discrepancy between the complexity of patients’ pain problems (influence to be active despite the pain, acceptance, and optimism as goals) and the provided treatment underscores the importance of adopting an integral vision of health, which emphasizes optimizing functioning despite the presence of chronic conditions [12].

Despite efforts to address chronic pain, including knee osteoarthritis, many Dutch patients still encounter inadequate treatment, leading to a phenomenon known as “medical shopping” [13,14,15]. This behavior, characterized by consulting multiple healthcare professionals ranging from six to over twenty in search of pain relief, underscores the need for improved collaboration and coordination among healthcare professionals to ensure comprehensive and patient-centered care [16]. This mismatch between the complexity of the patient’s pain problem and the provided treatment can be attributed to differences in knowledge and perspectives among healthcare professionals, policymakers, and the general public regarding chronic pain, referral, and treatment [17].

Recognizing the multifaceted nature of health experiences, there is increasing recognition of the importance of considering social determinants of health in addition to effective communication between healthcare professionals and patients is crucial for engaging patients in their care process and facilitating their participation in daily life activities [18]. To address the needs of patients with knee osteoarthritis, healthcare professionals must adopt an integral vision of health and collaborate with colleagues from various disciplines within a patient-centered care pathway. Although existing literature highlights that interprofessional collaboration among healthcare and allied healthcare professionals can positively impact patient outcomes in general, evidence specific to patients with osteoarthritis remains limited. Exploring this gap is important to better understand how such collaboration might enhance the care and daily life participation of individuals living with osteoarthritis. The International Classification of Functioning, Disability and Health (ICF) framework provides a valuable tool for exploring factors influencing patients’ participation in daily life activities, considering disease-related, personal, and environmental factors [19].

The ICF model is presented by the WHO as an instrument for analysis of functioning of the patient [19]. The model defines the underlying pathology, problems at the level of organ functioning, activity level, the potential for discussion optimal personal function and preventing further limitation. The ICF-framework considers the ability of an individual to participate in society with respect to their expectations, preferences and needs. Integration of disease-related, personal and environmental factors will empower patients, strengthen their self-efficacy, and provide them with greater understanding and control over their own health. Moreover, optimizing interprofessional teamwork through the ICF approach can enhance the quality of patient-centered care for knee osteoarthritis patients, a concept not yet fully integrated into current care pathways [9]. Thus, the ICF framework not only serves as an analytical tool but also underlines the competencies healthcare professionals need to work collaboratively across disciplines. Using the ICF-framework as an educational instrument can aid healthcare professionals in helping patients self-manage their disease and control their health, particularly during training programs for professionals with diverse backgrounds [20]. It can help to explain functioning in individuals with different diseases.

However, successful implementation of this integral vision of health in daily practice requires targeted training and continuous practice. The ICF model can be utilized in healthcare professional training programs to foster understanding of the relationship between disease and functioning and facilitate communication between professionals and patients, as well as among professionals from different disciplines [20,21]. Previous research indicates various factors influencing the adoption of an integral vision of health, including personal, environmental, organizational, and social factors [22,23,24]. Understanding these influences can enhance readiness for implementation and promote successful integration into clinical settings.

Knee osteoarthritis necessitates a comprehensive approach involving collaboration and coordination among various healthcare disciplines and professionals [25]. Insights are needed to improve communication between patients and healthcare professionals, ultimately aiming to enhance participation in daily life and opportunities for self-management in the patient’s care process.

Therefore, the aim of this study was to explore the perspectives of two key stakeholder groups: patients with knee osteoarthritis and healthcare professionals. Specifically, this study aimed to identify (1) what patients perceive as necessary healthcare for managing knee osteoarthritis, and (2) what healthcare professionals consider important to provide for integrated collaboration in care pathways.

## Methods

### Study design

A qualitative study was performed with patients with knee osteoarthritis and healthcare professionals. Ethical approval for this study was obtained by the Medical Research Ethics Committee of the Maastricht University Medical Center+ (MUMC+) (reference number: 2023–0157). All participants were recruited and interviewed between September 2023 and December 2023.

### Study Population

#### Patients with knee osteoarthritis

Potentially eligible patients were identified by the treating orthopedic surgeon of the Maastricht University Medical Center in the southern part of the Netherlands. Eligibility criteria were 1) patients with knee osteoarthritis, 2) aged ≥18 years, and 3) adequate understanding of the Dutch language. Patients for whom it was not possible to conduct an interview due to cognitive or medical problems were excluded. Information regarding the study was provided by the orthopedic surgeon during the first consultation in the hospital and a patient information form (PIF) was given to the patient. Interested patients received a patient information letter. Thereafter, the researcher contacted the patient to verify the willingness to participate and to schedule an interview after oral consent. Written informed consent was obtained before interviewing, and participants were explicitly informed that their participation was voluntary and that they could withdraw from the study at any time without any consequences. Participants were involved in the study by sharing their personal experiences and perspectives during semi-structured interviews. Their input informed the qualitative analysis. They were not involved in the study design or data interpretation.

#### Healthcare professionals

Healthcare professionals in the southern part of the Netherlands from various healthcare disciplines who were involved in the treatment process of patients with knee osteoarthritis varied from orthopedic surgeons, case managers, physician assistants, physical therapists, psychologists, general practitioners, and rehabilitation physician). Some of these professionals were actively involved in the knee network in South Limburg, a collaborative network of healthcare providers dedicated to the multidisciplinary care of patients with knee osteoarthritis. Names of these specialists were provided by the researchers (JV and TB). Healthcare professionals were informed and invited to participate in the study by e-mail by the researcher. Written informed consent from healthcare professionals was obtained at the start of the interview by the researcher after discussion of the purpose and requirements of the study.

### Data collection

Data was collected through one-to-one semi-structured interviews. For the patients, this was no later than 2 weeks after the appointment with the orthopedic surgeon in MUMC+. In time and place suited for each participant. Interviews were conducted via video consulting online or by telephone. One researcher (MV) conducted the interviews with patients and healthcare professionals. The sample size was based on obtaining sufficient information power to achieve the aims of the study [26]. The number of interviews intended to perform was based on inductive thematic saturation. Data collection was ended when additional interviews provide no new information. Saturation was considered when interviews did not lead to new themes. It was expected that approximately eight interviews would be required with patients and ten interviews with healthcare professionals.

### Content of the interviews

The interviews were conducted based on a semi-structured interview guide, which was created by MV, JV, IH, JN, GR, and using open-ended questions that initially defined the areas to be explored. Interview topics are shown in supplementary file 1. Initial topics discussed were chosen based on the Capability, Opportunity, and Motivation for Behavior (COM-B) model [27] to identify factors that influence collaboration within the care pathway of knee osteoarthritis with patients. The COM-B model posits that behavior is influenced by three components: capability (e.g., physical skills and knowledge), opportunity (e.g., environment and social norms), and motivation (e.g., habits and beliefs). This model encompasses all factors that affect behavioral change [27]. The topics utilized were need, self-direction, care received and experienced by the orthopedic surgeon for patients and care pathway and outpatient appointment with people with knee osteoarthritis, collaboration other disciplines and course requirements for healthcare professionals. Participants were asked about their expectations, preferences and beliefs about discussing the treatment and consequences of/or with patients with knee osteoarthritis. For patients, information also requested during the interview included patient characteristics and previous experiences with health problems.

### Data analysis

Data was collected according to the standards for reporting qualitative research checklist [28].

All interviews were recorded and transcribed verbatim by MV (physiotherapist with experience in musculoskeletal care). These transcripts were fragmented and open coded in NVivo. The open codes were divided into subthemes and themes using thematic analysis [29]. The first three interviews of each study population were independently fragmented, coded, and thematised by two researchers (MV and MM). Themes were discussed until consensus was reached by three researchers (MV, MM, and MG). These themes were used as a base for coding the other transcripts. Three peer debriefing sessions were held (MV, MM and MG) to critically discuss coding decisions and interpretations, promoting multiple perspectives and reducing individual bias.

### Results

#### Participants

A total of seventeen interviews were conducted with nine healthcare professionals and eight patients. Healthcare professionals were two orthopaedic surgeons, two physical therapists, one specialized nurse, one general practitioner, one physician assistant, one psychologist, and one rehabilitation physician. The mean age of the healthcare professionals was 42 (30–64) years. The mean age of the patients was 63 (49–73) years. All participant characteristics are summarized in Table 1. Final themes were described and summarized on a code tree (Table 2) The themes from the interviews are summarized in the text below.

**Table 1:** Characteristics of participating patients and healthcare professionals.


PARAMETERS		HEALTHCARE PROFESSIONALS (n = 9)	PATIENTS (n = 8)

**Sex** n (%)			

	Male	6 (67%)	3 (38%)

	Female	3 (33%)	5 (62%)

**Age** n (%)		42 (30–64)	63 (49–73)

30–39		5 (56%)	–

40–49		3 (33%)	1 (13%)

50–59		1 (11%)	2 (25%)

60–69		–	2 (25%)

70–79		–	3 (37%)

**Work** n (%)			

	Employed		4 (50%)

	Retired		4 (50%)

**Living situation**			

	Alone		2 (25%)

	Living together with partner		5 (62%)

	Living with children		1 (13%)

**Function**			

	Participant 1	General practitioner	

	Participant 2	Physician assistant	

	Participant 3	Orthopedic surgeon	

	Participant 4	Psychologist	

	Participant 5	Specialized nurse	

	Participant 6	Rehabilitation physician	

	Participant 7	Physiotherapist	

	Participant 8	Orthopedic surgeon	

	Participant 9	Physiotherapist	


**Table 2:** Code tree.


CODES	SUBTHEMES	THEMES

**Healthcare professionals**		

Diagnosis confusing to other healthcare professionals	Barriers to diagnosis	Diagnostics

GP refers too easily		

Orthopedic referral	Needs diagnostics	

Imaging for reassurance (MRI, X-ray)		

Ask about knee complaints		

Questionnaires as a supplement		

Expectations of patient diagnostics	Expectations diagnostics	

Challenge conversation about adjusting lifestyle	Barriers to conversation with patient	Conversation, information seeking, and care pathway

Patients need surgery		

Shift in care (social changes)		

Healthcare costs are high/handle with care		

Patient has different expectations		

Stigma referring dietitian		

Too little time to discuss lifestyle advice		

Mapping activity level	Patient’s experiences in conversation	

Discussing rules of life/reassuring the patient		

Keep doing sports		

Work sometimes negotiable		

Daily activities		

Lifestyle advice important		

Patient confidence	Promotional conversation with the patient	

Request for help central		

Discuss interventions clearly		

Patients are satisfied		

Refer to physical therapy		

Feels responsible for patient		

Knowledge/no knowledge of ICF	Experiences integral vision on health	Knowledge and skills of the integral vision on health

Information by referral		

Discussing patient barriers		

Yes/no time to discuss participation		

Asks for participation		

Patients see many healthcare professionals	Barriers integral vision on health	

Lifestyle advice belongs to another healthcare professional		

Little time for other topics of conversation		

Knowledge expertise healthcare professionals	Collaboration needs	Perceived collaboration and support from healthcare professionals

Referral to other healthcare professionals must be clear		

Generic work		

Time investment	Barriers to cooperation	

Contact with healthcare professionals helpful/difficult		

Finding each other difficult		

Not knowing what the other person does/expertise		

Knowledge of each other’s expertise	Collaboration needs	

Measuring quality of care is necessary		

Accreditation required	Education expectations	Conditions of training for healthcare professionals

Healthcare professionals work more generically		

Share substantive knowledge	Need training	

Gain knowledge of the whole care path		

More interdisciplinary work		

Expand the network of healthcare professionals		

Time investment	Wishes for education	

**Patients**		

Need for diagnostics (MRI, X-ray)	Needs diagnostics	Diagnostics

Broad view by orthopedist		

Diagnosis confusing to other healthcare professionals	Barriers to diagnosis	

Multiple visits to the doctor are necessary	Experiences in diagnostics	

Orthopedic referral		

Knee complaints		

Would like MRI/diagnosis	Expectations diagnostics	

Challenge of adjusting lifestyle	Experiences conversation	Conversation, information seeking, and care pathway

Satisfied/dissatisfied with healthcare professional		

Discuss participation/work		

Everything is negotiable		

Asking for help is central		

Purchased tools myself		

Activity level	Need conversation	

Physical therapy referral		

Discuss rules of life		

Advice regarding participation/sport		

What interventions are possible		

Intervention advice		

Patient trust about healthcare professional	Barriers conversation	

Does not want a dietitian referral		

Anxiety intervention		

Healthcare costs		

Different expectations than healthcare professional		

Discuss rules of life	Information provision	

Lifestyle advice		

Seek help yourself online		

Different healthcare professionals say something different	Barrier information	

Broad view by orthopedist	Need for an integral vision on health	Knowledge and skills of the integral vision on health

Lifestyle/participation advice		

Expectation of knee diagnosis	Experiences integral vision on health	

Orthopedic surgeon knows what he is saying		

Physical therapist paid attention		

Need for an X-ray		

Referral to healthcare professional is well arranged	Experiences of healthcare professionals	Perceived collaboration and support from healthcare

General practitioner refers late	Barriers to healthcare professionals	

Physical therapy costs		


**Abbreviations**: ICF = International Classification of Functioning, Disability and Health, MRI = Magnetic resonance imaging.

#### Patients

##### Diagnostics

Patients expressed a strong need for clarity regarding their complaints and sought a diagnosis. This need was frequently linked to a desire for specific imaging techniques, such as MRI scans or X-rays, to confirm the diagnosis. Regardless of the implications of the diagnosis, most patients sought a clear understanding of their medical situation. Patients found it confusing that different healthcare professionals attributed different causes to their knee complaints. Additionally, patients reported that receiving a diagnosis could make them accountable to those around them and could facilitate modifications to their exercise behaviours.

*“My expectations from the orthopaedic surgeon were, well, my knee is bothering me. Is there something different going on? If there is nothing wrong with the knee, maybe it is due to my changed gait pattern after my total hip prostheses?” Patient 2*

*“With an MRI you know for sure what it is. I find it a bit too easy to say without an MRI: Then that’s probably it… I’m not going to say that the general practitioner is holding me back, but I do feel the need for diagnostics to be able to say, there is also a problem here osteoarthritis in or there is an irritation in the joint or that they can at least give me some guidance, then you should no longer do certain activities.” Patient 3*

##### Conversation, information seeking, and care pathway

Patients consistently expressed a need for trustworthy information on the internet, particularly regarding appropriate physical activity levels. Patients sought confirmation regarding what constituted beneficial versus harmful movements, emphasizing the critical role of the platform in guiding patient behaviours and choices. Patients indicated that they had confidence in healthcare professionals, who almost always addressed their requests for help. Most patients reported receiving lifestyle advice from orthopaedic surgeons, such as recommendations regarding work and walking. They found referrals to other healthcare professionals, such as physical therapists, acceptable.

*“I liked the fact that the orthopaedist indicated that it is a good idea to start with physical therapist conservatively and that I should come back in three months to see what effect it has on my knee complaints.” Patient 6*

In the Netherlands, access to physical therapy often requires supplementary health insurance, resulting in additional expenses for individuals’ healthcare budgets. Several patients mentioned that the cost of care served as a barrier to attending physical therapy sessions. Some patients who received referrals for dietetics did not perceive the added value of seeing a dietitian but expressed willingness to pay more attention to their diet. Patients indicated that they could make their own decisions regarding the interventions or conservative treatment options offered (such as physical therapy or dietary advice) and that they could also discuss this with healthcare professionals. Healthcare professionals perceived individuals with osteoarthritis as reluctant to follow advice regarding weight loss. This reluctance was interpreted as a lack of motivation or limited confidence in the effectiveness of lifestyle changes.

##### Knowledge and skills of the integral vision on health

Patients expressed concerns about healthcare professionals focusing exclusively on their knee issues, potentially overlooking other factors such as back and foot pain or vascular problems contributing to their symptoms. They underscored the importance of addressing their comorbidities’ needs and appreciated it when all caregivers listened to their physical complaints, specific requests, and concerns that might influence their knee complaints.

*“I thought I could ask all the questions I had, but afterwards I thought, are we looking broadly enough? Are my complaints really due to knee osteoarthritis?” Patient 3*

##### Collaboration with other healthcare professionals

Some patients indicated that the general practitioner referred them adequately to the orthopaedic surgeon, other patients indicated that several appointments with the general practitioner were necessary and that they had to insist on a referral to the orthopaedic surgeon.

*“I often think of the procrastination in general practitioners. They say: just go home and if the complaints persist, come back again. But before I go to the doctor, I have already tried everything that I think could help to prevent the complaints.” Patient 4*

Patients indicated referral to the physical therapist was well-coordinated, and they adhered to the advice provided by healthcare professionals through the referral. Patients also mentioned encountering conflicting statements regarding their knee complaints from different healthcare professionals, including the physical therapist, general practitioner, and orthopaedic surgeons. Patients reported facing duplicate examinations related to their knee complaints and the resulting consequences.

### Healthcare professionals

#### Diagnostics

Healthcare professionals stated that they encountered challenges in meeting patients’ expectations, particularly regarding the prevailing beliefs patients held about the causes or significance of knee osteoarthritis. On one hand, healthcare professionals believed that diagnostic imaging was crucial for sufficiently informing and reassuring patients. On the other hand, some healthcare professionals such as the orthopaedic surgeon and rehabilitation physician proposed that additional imaging was not strictly essential for diagnosis. Healthcare professionals such as the general practitioner, orthopaedic surgeons, and the physiotherapists mentioned that they occasionally pursued further diagnostics to fulfil patients’ expectations for clarity regarding their complaints and the necessity for a diagnosis.

*“What I notice is that most patients don’t really want to have surgery, but what they especially want to know is a clear diagnosis so that they know what they can do about knee complaints.” Healthcare professional 3*

#### Conversation, information seeking, and care pathway

One of the primary challenges healthcare professionals indicated facing was navigating the sensitivity and potential stigma associated with discussing overweight with patients. While some healthcare professionals (the orthopaedic surgeons and physiotherapists) mentioned advising weight loss for various health reasons, patients often expressed reluctance. Healthcare professionals acknowledged the challenge of ensuring that patients understood and applied the advice and education promoting self-management. They indicated that patients often did not immediately grasp information about a healthy lifestyle. Despite professionals’ concerns about patient comprehension, they noted that patients generally expressed satisfaction with the information received (such as www.thuisarts.nl).

*“I have become a fan of the osteoarthritis selection card on www.thuisarts.nl. You can present this to patients. A heading could be added about the importance of continuing to exercise and its health effects.” Healthcare professional 1*

One challenge highlighted by healthcare professionals was the inability to monitor whether patients act upon the advice given. This limitation presented practical challenges in assessing the effectiveness of recommendations and customizing care plans to individual patient behaviours and preferences.

#### Knowledge and skills of the integral vision on health

Healthcare professionals, such as the orthopaedic surgeon and general practitioner emphasized the importance of addressing daily life consequences, such as difficulties in performing work or daily activities, but they highlighted challenges in allocating sufficient time for these discussions with the patient. While the impact on daily life was routinely addressed by healthcare professionals, the depth and frequency of these conversations varied, often limited by time constraints rather than a lack of recognition of their significance. To overcome time constraints and ensure comprehensive care, all healthcare professionals underscored the importance of collaboration among interdisciplinary team members. They also mentioned that discussing lifestyle was difficult because it was unknown what options were available regarding a lifestyle intervention, and they did not know where to refer patients to.

*“I consider lifestyle to be one of the most crucial factors, as it often presents an opportunity for improvement in nine out of ten patients. It can be frustrating not to offer them something concrete. For example, referral to physical therapy networks can sometimes be a cumbersome process.” Healthcare professional 8*

Healthcare professionals indicated that patients’ requests for help were usually aimed at reducing knee pain, and the intervention they recommended and implemented was therefore typically aimed at alleviating knee complaints rather than focusing on an integral vision of the patient’s health. Half of the healthcare professionals such as the rehabilitation physician and physiotherapist acknowledged familiarity with the ICF framework, while others admitted to having limited knowledge or facing challenges in explaining its principles. Half of the healthcare professionals, such as the rehabilitation physician and physiotherapist, acknowledged familiarity with the ICF framework, while others admitted to having limited knowledge or facing challenges in explaining its principles.

#### Perceived collaboration and support from healthcare professionals

Interprofessional collaboration refers to the process by which healthcare professionals from diverse disciplines work together to provide patient-centred care. Healthcare professionals indicated that efficient and effective referral systems, such as regional referral cards, were critical to optimizing patient outcomes and ensuring coordinated care among healthcare professionals. Medical specialists from MUMC+, general practitioners, physical therapists, and exercise coaches worked closely together in a knee network. Healthcare professionals underscored the importance and added value of the existing knee network of physical therapists in South Limburg and highlighted its role in facilitating referrals and streamlining patient care. However, some professionals (e.g., general practitioner physician assistant, specialized nurse, and psychologist) mentioned that they were not familiar with this network. Healthcare professionals expressed a need to expand their knowledge of care pathways for knee osteoarthritis beyond their specific roles. They mentioned that increasing interdisciplinary knowledge and expertise would enable them to make informed referrals, ensuring patients receive targeted and comprehensive care tailored to their unique needs. Continuity of care and access to comprehensive information were mentioned as essential for monitoring patient progress, evaluating outcomes, and adjusting treatment plans. When receiving referrals, healthcare professionals expressed a need for clear and detailed transfer of healthcare information, including comprehensive patient histories, diagnostic findings, treatment plans, and follow-up recommendations.

*“So I think that we as healthcare professionals should agree in a way that we can share information with each other a little more easily without drowning out privacy. But we can look at what is in the MRI report, what is in the letter of the general practitioner, what is in the websites and how are we going to continuously coordinate this with each other in a natural working method.” Healthcare professional 8*

Healthcare professionals occasionally expressed reservations about the expertise of other professionals within the referral network. Addressing these concerns entailed fostering trust, promoting collaboration, and establishing mechanisms to evaluate and ensure consistent quality of care across professionals.

*“I also discovered that a large number of my colleagues, both old and young, with much and little experience, that their knowledge regarding the diagnosis of knee osteoarthritis can sometimes be insufficient. And this means that a referral to the second line is made quite quickly.” Healthcare professional 9*

Healthcare professionals noted discrepancies among themselves in the information conveyed to patients, underscoring the necessity for standardized protocols, evidence-based practices, and clear communication strategies to guarantee consistent messaging and enhance patient understanding and engagement. Furthermore, healthcare professionals indicated that patients interact with multiple healthcare professionals, each possessing their own expertise and perspectives.

#### Conditions of training for healthcare professionals

All healthcare professionals mentioned that education is needed to effectively integrate the integral vision of health into clinical practice, thereby enhancing collaboration among diverse healthcare disciplines and providing clear information to patients. This education aimed to facilitate successful implementation and enhance cooperation within healthcare settings. The necessity for an integrated approach to training and the establishment of a network as an outcome were also cited as prerequisites for organizing such training. Concerning the practical aspects of the training, healthcare professionals believed that accreditation for training should be mandatory. Additionally, due to scheduling constraints and time limitations, they suggested that training should consist of approximately three half-day sessions per year.

*“I would like to know from other healthcare professionals what they can offer patients with knee osteoarthritis so that I can refer better and so that I also know where I can refer by expanding my network of healthcare disciplines.” Healthcare professional 7*

## Discussion

The aim of this study was to conduct a stakeholder analysis to identify the perspectives of patients and healthcare professionals and uncover factors that influence integrated collaboration within the care pathway for patients with knee osteoarthritis. Some patients expressed concerns that healthcare professionals did not comprehensively consider the underlying causes of their knee issues. Patients indicated satisfaction with the care received for knee osteoarthritis and emphasized the need for a clear diagnosis. Healthcare professionals, especially the general practitioner and orthopedic surgeons, cited a lack of knowledge about the integral vision of health in knee osteoarthritis and insufficient time with patients as barriers to discussing lifestyle factors. Healthcare professionals highlighted the need for an interdisciplinary approach to training to implement the integral vision of health and to build networks as outcomes. Healthcare professionals mentioned that they often applied diagnostics to meet patients’ expectations. They were satisfied with the knee network in their region but emphasized the need for collaboration between different disciplines to be aware of each other’s expertise, thus improving referrals and ensuring patients received targeted and comprehensive care tailored to their unique needs.

In this study, patients felt understood and heard, which increased their trust in their healthcare professionals. However, patients noted that healthcare professionals did not always consider their complaints broadly enough, as there were other medical complaints besides knee osteoarthritis. An integral vision of health approach to care can enhance communication and the relationship between caregivers and patients [20,30]. Having the right skills and knowledge to apply an integral vision of health to patient care is essential. It is important that patients should be viewed more comprehensively as persons experiencing a health issue, thereby encompassing not only the medical dimension but also considering the context of the person and their capacity for self-management within the given circumstances. Previous research among healthcare professionals found that implementing an integral vision of health using the ICF-framework positively contributed to learning about other professions, mentor experiences, treating the whole person, and improving team effectiveness [20,31].

This study found that patients had a strong need for a medical diagnosis through imaging examinations. Seeking a diagnosis may be influenced by the stigma surrounding illness and symptoms [32]. Previous research showed that half of patients with chronic pain feel stigma and discrimination from those around them because of their pain [33]. Seeking a diagnosis is often influenced by the need for understanding and acceptance in their environment [34]. Stigma can have a significant impact on patients. It can lead to fear of rejection, social exclusion and even avoidance of seeking necessary care [32,33,34]. Patients still regularly expect healthcare professionals to diagnose, prescribe treatments and provide guidelines aimed at full recovery from symptoms [25]. However, a diagnosis of osteoarthritis indicates a chronic disease where full recovery is not an option [11]. This research shows that obtaining a correct diagnosis is a complex process for some patients, requiring them to consult multiple healthcare professionals before getting a clear picture of their health status. In this study, patients mentioned facing duplicate examinations and having to repeat their story each time they saw a new healthcare professional, which can be costly and time-consuming for both patients and the healthcare system [35].

By considering the whole person and not just the disease, healthcare professionals can positively impact their patients’ lives and contribute to a healthier society [21]. Embracing a holistic approach in chronic disease management is crucial for maximizing healthcare evolution’s potential in managing chronic conditions. Prioritizing functionality and personal values over symptom relief embodies patient-centred care principles. Chronic disease management emphasizes promoting resilience, enabling individuals to thrive despite their health problems. This research shows that healthcare professionals still often avoid discussing factors that can be modified by the patient in the context of health on the patient’s limitations on their daily life caused by pain. An integral vision of health can facilitate preventive measures and early interventions, leading to cost savings by preventing more serious health problems and reducing the need for multiple healthcare consultations to assess health problems [36].

In this study, all healthcare professionals expressed the need for interdisciplinary meetings to gain knowledge of each other’s expertise in treating patients with knee osteoarthritis. There is also a need to expand the network of healthcare professionals to work more integratively. To determine if patient care is complex, an integrated approach is necessary, considering not only physical manifestations but also psychological, social, and environmental factors. Assessing patient care requires an integrated approach, considering physical, psychological, social, and environmental factors. A patient-centred, integrated approach is crucial in healthcare decision-making. This shift from monodisciplinary to interdisciplinary work focusing on the integral vision of health is supported by the WHO. Healthcare professionals should provide patients with the necessary information and guidance tailored to their wants and needs while being attentive to patients who lack self-direction skills [21]. Recent insights suggest that patients increasingly value aspects of the healthcare experience related to improving functioning and health among healthcare professionals [30,37]. Healthcare professionals in this study expressed the need for educational initiatives to effectively integrate the integral vision of health into the care of patients with knee osteoarthritis into clinical practice. Therefore, interprofessional education is important for developing the necessary competencies for working in interprofessional teams across professional boundaries [38].

### Strengths and limitations

This stakeholder analysis considered the unique characteristics of the local context, including the hospital’s specific care pathways for patients with knee osteoarthritis. However, as with any qualitative study, the positionality of the researchers is an important factor to consider when interpreting the results. The interview guide’s compilation by an interdisciplinary project group and the analysis by a physical therapist and a research assistant may have influenced the formulation of questions and interpretation of responses. In this study, the interview guide was developed by an interdisciplinary project group, which included professionals from various healthcare fields, including physical therapy. Additionally, the analysis was conducted by a physical therapist and a research assistant, which may have impacted the interpretation of responses, particularly with regard to physical therapy-related aspects of care. Acknowledging these potential influences allows readers to better understand the lens through which the data was analyzed and the way interpretations were made, and helps to position the findings within a broader context.

The geographical restriction to patients and professionals in a hospital in a small part of the Netherlands may limit the generalizability of the findings. Future research might consider broadening the geographic scope and including participants from different regions to obtain a more diverse and representative sample. The development of the integral vision of health aims to support personalized and patient-centred care in clinical settings. Therefore, it is essential to train current healthcare professionals in good interprofessional collaboration and to incorporate the integral vision of health into their practice. Additionally, this approach should become a regular part of training for future healthcare professionals.

The development of the integral vision on health is aimed at initiating support in clinical care, providing opportunities for personalized and patient-centred care. Therefore, it is imperative to provide current healthcare professionals with training in effective interprofessional collaboration and in integrating the patient with an integral vision of health. Furthermore, this approach should be incorporated as a standard component of training for future healthcare professionals.

## Conclusion

This stakeholder analysis provides valuable insights into the perspectives of both patients and healthcare professionals regarding integrated collaboration within the care pathway for knee osteoarthritis. Patients articulated a desire for healthcare professionals to comprehensively consider underlying causes while emphasizing the importance of receiving clear diagnoses. Overall, patients expressed satisfaction with the care they received. Healthcare professionals identified various barriers, including limited knowledge of the integral vision on health, time constraints, and the necessity for interdisciplinary training to effectively promote an integral vision on health. They underscored the significance of interdisciplinary collaboration to improve referrals and ensure tailored, comprehensive care for patients. The development and implementation of an integral vision on health are crucial for fostering personalized and patient-centred approaches in clinical practice. This requires continuous training for current healthcare professionals in effective interpersonal collaboration and the integration of integrated health paradigms. Furthermore, incorporating this approach into the curriculum for future healthcare professionals is vital to its continued advancement and widespread adoption across healthcare settings.

## Data Accessibility Statement

The data underlying this article will be shared on reasonable request to the corresponding author.

## Additional File

The additional file for this article can be found as follows:

10.5334/ijic.8969.s1 Supplementary file 1. Interview topic guide.
