# Supplementary material for: Enhancing Collaboration and Integrated Vision on Health: Key Strategies for Addressing Knee Osteoarthritis
Source: Int J Integr Care. 2025 Sep 16;25(3):28. doi: 10.5334/ijic.8969 (PMC12447795; doi:10.5334/ijic.8969)
Supplement: Supplementary file 1. — Interview topic guide. [file ijic-25-3-8969-s1.pdf]

Supplementary files

Supplementary file 1. Interview topic guide

Patients

Needs

1. With what need/question did you go to the general practitioner?
    a. What complaints do you have?
    b. Since when have you been experiencing these complaints?
    c. What treatment options were you hoping to receive?
    d. What did you expect from the conversation with the orthopedist?
    e. What are/were your expectations and wishes regarding treatment and care?
2. How do your complaints affect your daily life? (Activity such as work, hobbies, ADL).
3. Do you feel that your request for assistance was central to the care provided by the orthopedist/other healthcare professionals? (How, when, and where did that happen: further questions in the areas of positioning, activities and participation).

Self-direction

4. What have you started doing differently since you started having complaints?
    a. Which other healthcare professionals have you consulted about your complaints? What did you gain from their help (and how did you feel about it)? What guidance was helpful to you? If you have been to another healthcare professional, how did you end up with the orthopedist?
5. What could help you/what could you do yourself to function better despite the obstacles and complaints? What do you need?

Received and experienced care from the orthopedic surgeon

6. Were there things discussed that surprised or pleased you? If so, what?
7. What information did you receive from the orthopedic surgeon about knee osteoarthritis?
    a. Do you now know what you can and cannot do regarding strain/load on your knee?
    b. Do you now know enough to get started yourself?
8. What information do you need to resume your daily activities?
9. What could you discuss with the care professional?
10. What did you think of the treatment recommended by the orthopedist? What did you think of the orthopedist's explanation?
11. Did the orthopedic surgeon provide you with advice or treatment options that you could decide on yourself?
    a. Were you able to decide for yourself which treatment options best suited your request for help?
    b. Did you have enough information, or did you need more information?
12. What advice has the orthopedic surgeon given regarding exercise, sports, and nutrition?
13. What other healthcare professionals have you been referred to? For example, a physical therapist, dietician or psychologist?
14. Is there anything else you would like to say that is important for your treatment?

Healthcare professionals

Care path and outpatient appointment with people with knee osteoarthritis

1. Can you tell us what a first appointment with people with knee problems looks like?
2. Can you outline the care path for someone with knee osteoarthritis?
    a. What are you satisfied with?
    b. What could be different/better?
3. How satisfied do you think people with knee osteoarthritis are with the care they currently receive in this pathway?
4. How could you improve care yourself or in collaboration with other healthcare professionals to ensure that the patient is more satisfied?
5. After the conversation, what do you know about the person who came with knee osteoarthritis?
6. Have you heard of ICF; what do you think? (If necessary, explain ICF or test what the healthcare professional knows about it).
7. If you think back to the last consultation hour you had:
    a. What was the request for help from this person with knee osteoarthritis?
    b. What were the important daily activities of this person with knee osteoarthritis in which he/she was hindered by the knee osteoarthritis?

    c. What physical capabilities does this person with knee osteoarthritis need in his/her work situation?
    d. How do you think this person with knee osteoarthritis can put the advice into practice?
    e. What are the most common requests for help from people who come to your consultation hours with knee osteoarthritis?
8. What do you think about discussing participation with this person with knee osteoarthritis? To what extent do you discuss ADL, expectations, goals, treatment options, self-management with the person with knee osteoarthritis?
    a. What is the added value of this information with regard to the treatment?
    b. What is an obstacle to discussing this?
9. What do you think are the main challenges and complexities in providing care to individuals with knee osteoarthritis?
10. How much time would you need in the consultation room to ask questions about the impact of knee osteoarthritis on daily life?
11. To what extent do you discuss daily functioning with the person with knee osteoarthritis (and what works and what does not)?
12. What do you need to ensure that you pay attention to the impact of knee osteoarthritis on daily life?
13. From your perspective, what are the specific needs and wishes of people with knee osteoarthritis regarding their treatment and care?

Collaboratie with other disciplines

14. What does your collaboration look like with colleagues within and outside the hospital regarding knee osteoarthritis?
    a. If so, with whom and what does that collaboration look like?
    b. What is going well, and how can you tell?
    c. Are you missing something here? What could be better?
    d. What do you think ideal collaboration looks like?
    e. What is required for this?
    f. What could another healthcare professional (such as physical therapist, dietician, psychologist) do regarding the care you provide for people with knee osteoarthritis?
15. How do you currently work with other healthcare professionals to properly guide and monitor people with knee osteoarthritis? Are there specific practices or strategies that you find particularly effective?
    a. Which healthcare professional do you sometimes refer to?
    b. What do you see as the most important factors promoting collaboration in the care pathways for knee osteoarthritis?
    c. What obstacles do you experience that prevent effective collaboration?
    d. What types of resources or support would you find helpful to promote collaboration and integrated care for patients with knee osteoarthritis?
    e. What do you expect from what someone else does when you seek cooperation? What contribution can someone make to healthcare or what responsibilities does that other person have?
    f. To what extent do you feel responsible for the disease and the impact of the disease on people with knee osteoarthritis?
    g. What do you think falls under your responsibility and where are the boundaries?
    h. What can you contribute to providing care through collaboration (a matter of trust and mutual support)?

Course conditions

16. What should a course focused on the integrated vision look like to follow it?
    a. What should it bring you?
    b. What should a course look like for you to ensure that it is feasible, and the content can be applied?
17. What is the added value for you of collaboration with the exercise center/primary care?
18. Do you ever refer people with knee osteoarthritis to physical therapy or a dietician?
    a. If so, how often, where, when?
19. How does referral to, for example, a physical therapist work?
    a. What should you do about this yourself?
20. Do you ever ask someone with knee osteoarthritis if he/she understood you?

Are there anything else you would like to say that is important to you for providing optimal care and working with an integrated vision of health?
